# Real Workload-Situated Training in COVID-19 Prevention of General Practice Residents in China: A Situated Cognition Study

**DOI:** 10.3389/fpubh.2021.765402

**Published:** 2021-11-18

**Authors:** Rao Xin, Luo Li, Su Qiaoli, Wang Xingyue

**Affiliations:** ^1^Department of General Practice, West China Hospital, Sichuan University, Chengdu, China; ^2^Institute of Hospital Management, West China Hospital, Sichuan University, Chengdu, China; ^3^Institute of Service Management, Business School, Sichuan University, Chengdu, China; ^4^Department of Graduate Medical Education, West China Hospital, Sichuan University, Chengdu, China

**Keywords:** situated cognition, workload, COVID-19, general practice, resident

## Abstract

**Objective:** The participation of general practice (GP) residents in COVID-19 prevention and control tasks touched workload participation in public health and disease prevention and control and was also a rare, valuable training experience for the residents and research material for medical education. This experience contributed to the understanding of three key points: First, was the content of the COVID-19 prevention task suited to them, or did it overload them in the present? Second, their competence in the COVID-19 prevention task reflected whether the early medical school training was sufficient or not. Third, what can be drawn from this study to promote public health training in the future? This study aimed to explore these issues by conducting a real epidemic situated training (REST) program.

**Methods:** A situated cognition study was designed that included situational context design, legitimate peripheral participation, and the construction of a community of practice. The Task Cognitive Load Scale (NASA-TLX Scale) and self-developed questionnaires were adopted to conduct a questionnaire survey of resident doctors in a GP training program from West China Hospital of Sichuan University, and 183 questionnaires were collected. SPSS 23.0 statistical software was used for the statistical analysis of data.

**Results:** The NASA scale showed that the intensity of field epidemic prevention and control (training) was tolerable. In particular, there was statistical difference in the cognitive load intensity of training before and after the epidemic occurred at different time points (*P* < 0.05). This shows that they were early trained and well-prepared before sudden outbreak of the COVID-19. Before the outbreak of the epidemic, the public health knowledge and training received came from undergraduate education (83.16%), early residents program training (69.47%), online self-study (49.16%), and continuing education (20.53%).

**Conclusion:** Former medical school education and training at the regulatory training stage have a good effect and enable residents to master the skills required for epidemic prevention and control and to physically and mentally prepare for the task. After this stage, epidemic prevention and control training in real situations will make important contributions to the self-assessment and performance improvement of public health training.

## Introduction

The sudden outbreak of the COVID-19 pandemic highlighted the importance of competent community prevention as the front line of epidemic prevention and control and obliged practitioner residents to become part of such teams. The involvement of residents touched workload participation in public health and disease prevention and control and was also a rare and valuable training experience.

The main aim in the study was to assess whether the GP residents had the ability to respond to public health emergencies and well-prepared mentally and physically. In the study, the REST program (real epidemic situated training) was conduct to reveal the fact. On one hand, the REST program was a training program, on the other hand, the program was a mission in which a nature assessment was taken to evaluate whether the residents had the ability to respond to public health emergencies by evaluating their performance and workload item. The scale should be in acceptable range.

In the program, the study fully followed the process of situated cognition theory. Learning evolves within the simulation environment from the interactions of (a) activity, (b) people, and (c) prior knowledge that is brought to the situation. When knowledge and learning are theorized in terms of representations stored and processed in the mind, empirical and theoretical developments in very different scholarly disciplines have led to the emergence of the situated cognition hypothesis, which consists of a set of interlocking theses: cognition is embodied, fundamentally social, distributed, and enacted and often works without representations (1). A number of theoretical perspectives (2–4) have suggested that learning is socially situated, and cognitive development occurs through relations between human interactions and the social or cultural context of everyday activities, thereby laying the groundwork for challenging the assumption that skills learned in the classroom can simply be transferred to a practical environment (5). In other words, students, as newcomers, are guided by more experienced faculty and clinicians to actively take part in the learning process, which allows them to construct their own knowledge based on relationships with other learners, activities, environmental cues, and the social organization of learning events. Situated cognition has been established as a useful framework for understanding and guiding teaching and learning processes in medicine education and training (6).

Simulation-based learning is a dynamic process that can be used to build a community of practice where learners can observe and participate in meaningful realistic encounters to build their skills and understanding (7–9). Moreover, they can gain insights and feedforward, which is described as the opportunity to envision future activities after mastering certain skills and behaviors (5, 10).

The outbreak of the COVID-19 pandemic conducts a real situation for the GP resident to participate in the public health task (11, 12). This real situation may be a once-in-a-century experience that cannot be replicated. The real epidemic situated training (REST) program during COVID 19 has many implications for the field public health training. This study aimed to explore the research on the training content for residents as well as their cognitive load and abilities improvement at different time points (before, during, and after the outbreak) in a real situation, to use in the study of the resident training process.

## Methods

### Participants

In the study, the full sample population survey was conducted, 183 questionnaires who enrolled GP training program in West China Hospital of Sichuan University were finally collected.

### Design

Situated cognition is classified into studies of situational context design, legitimate peripheral participation, and the construction of a community of practice (1).

### Situational Context Design

Following the outbreak of the COVID-19 epidemic, China initiated a first-level response to the public health emergency (13). The main purpose of standardized training is to train qualified grassroots general practices, and an important part of the training content concerns responding to public health emergencies. Community health service centers played an extremely important role as the first line of defense in the prevention and control of the COVID-19 epidemic. They also provided real training scenarios for scientists' basic epidemic prevention practices. Each grassroots practice base must be reinforced according to the epidemic prevention and control situation. The teaching, training, and practice of infectious disease prevention and control knowledge and skills will use the completed training practice as an important basis for assessment of the process.

a. Pre-vocational training (before they tackle the real task) and assessment
  1. National Basic Public Health Service Standards—Service Standards for Reporting and Handling of Infectious Diseases and Public Health Emergencies
  2. Knowledge of epidemic prevention and control, hospital infection prevention and control, personal protection skills, etc.
  3. Introduction to each point of the institution's epidemic prevention and control work (layout, process, work content, specifications, etc.)
  4. Written examination to assess knowledge of epidemic prevention and control
  5. Standard patient (SP) assessment, prevention and control deployment process.
b. Materials guarantee

Provide personal protective materials (such as masks, gloves, protective clothing, isolation clothing, etc.) required for training participants at different points in the work.

### Legitimate Peripheral Participation

“legal peripheral participation” means learners should enter the scene, start at the edges, and continue to advance gradually and more deeply to master the core essentials and exert their subjective initiative in practice (7, 14). The acquisition of practical skills by students is a gradual process that does not occur overnight and requires the joint effort and participation of teachers and students. Online virtual simulation experiments can provide modular learning, and learners can complete different learning tasks according to their own learning needs (15–17). Through activities ranging from easy to difficult, teachers can inspire or induce students to actively explore in real or virtual experimental environments to give full play to the students' subjective initiative and in-depth studies. When students have cultivated themselves with competence, support and help from teachers can be appropriately reduced. According to the prevention and control work needs, the West China Hospital of Sichuan University arranges training for trainees to rotate in various epidemic prevention and control positions in the community health center and participate in prevention and control work. In addition, it arranges special personnel to be responsible for teaching. Under the guidance of the senior physician, the resident gradually becomes familiar with the work process.

The real epidemic situated training (REST) program during COVID-19 was conducted as following.

1. Pre-inspection and triage: participate in pre-inspection and triage, become familiar with the work process and specifications, master the main points of pre-inspection (temperature measurement, epidemiological history collection, simple inquiry of clinical symptoms, full use of general practice thinking) and become familiar with the triage process (outpatient clinic, general outpatient clinic, fever clinic, and referral).
2. Medical observation of quarantined persons:
  1) Home quarantine personnel: participate in the medical observation work of home quarantine personnel temperature measurement and health and traveling history inquiries
  2) Observation and isolation personnel: participants in medical observation on the personnel under centralized observation
  3) Concentrated residents: trainees of the plan can participate in the duty and medical observation of the hotels where people from Hubei (or other areas with a high rate of infection) are concentrated in Chengdu.
3. Participation in epidemic prevention and control and the daily work of the family medicine team:
  1) Education on epidemic prevention and control knowledge of contracted residents conducted through online methods such as online telephone and online software
  2) Chronic disease follow-up and health management during the epidemic period
  3) Practice of group epidemic prevention and elimination in enterprises, institutions, and schools
  4) Prevention and control of hospital infection and practice of occupational protection
  5) Other public health work in the community.
4. According to the specific situation, arrange for training students to participate in fever clinics, general clinics, children's health care, Chinese medicine, and rehabilitation and record the workload.

### Construction of a Community of Practice

Contextual cognitive learning theory emphasizes the construction of a community of practice. Teachers are not only lecturers of knowledge, but also the promoters of knowledge, the learning partners of students, and the promoters of communication among members of the community (18, 19). While teaching knowledge and cultivating abilities, experimental teaching also emphasizes the establishment of good thinking habits for students. Based on the real clinical cases, students can restore the complex context of knowledge generation and development under the guidance of teachers and obtain more flexible knowledge which adapt to the real situation. Through different case situations, students will have more opportunities to solve problems and apply the theoretical knowledge they have learned to their practice. In the community, there is interaction among individuals and between individuals and the environment. This learning process is a process of interaction, communication, and dialogue. Participants share resources, respect, and trust one another. In the practice community, the identity of the learner is continuously reproduced, and the learner moves from being a bystander to a participant and, finally, to a demonstrator of mature practice. In other words, they change from being a legitimate marginal participant as a novice to being a core member of the community as an expert step by step (20, 21).

## Cognitive Load Scale (NASA-TLX Scale) and Questionnaires

The NASA Task Load Index (TLX) is a popular technique for measuring subjective mental workload (22–24). It relies on a multidimensional construct to derive an overall workload score based on a weighted average of ratings on six subscales: mental demand, physical demand, temporal demand, performance, effort, and frustration level (23, 25). The Object Cognitive Load Scale (NASA-TLX Scale) and self-developed questionnaires were adopted to conduct a questionnaire survey of resident doctors who were in a GP training program in West China Hospital of Sichuan University, and 183 questionnaires were finally collected. The NASA-TLX Scale and self-developed questionnaires were finally merged into one questionnaire. The questionnaire prompts were as follows:

1. Have you participated in community epidemic prevention work?
2. When you participated in the epidemic prevention work, your identity was [single-choice question]
3. You participated in the prevention and control of the epidemic several months after the outbreak. [single-choice question]
4. In which community health service centers did you participate in epidemic prevention and control? [multiple-choice question]
5. The main contents of your participation were [multiple-choice question]
6. The training you received before taking up your post-included [multiple-choice question]
7. The teacher who provided you with pre-vocational training came from [multiple-choice question]
8. Degree of mental and cognitive stress when you participated in prevention and control work [single-choice question]
9. When you participated in the prevention and control work, did you have a heavy mental and physical burden? [multiple-choice question]
10. When you participated in the prevention and control work, did you have a strong sense of time urgency? [multiple-choice question]
11. When you participated in the prevention and control work, how hard did you need to work compared with other work? [single-choice question]
12. Did you feel pressure or worry during your participation in the prevention and control work? [multiple-choice question]
13. What is your satisfaction score with the REST program?
14. Are you willing to participate in epidemic prevention and control and continue to receive relevant training? [multiple-choice question]
15. Before the outbreak, the public health training you received included [multiple-choice question]
16. Before the outbreak, the public health knowledge and training you received came from [multiple-choice question]
17. If you are responsible for the prevention and control of an epidemic situation in a community in the future, the level you can achieve is [single-choice question].

## Results

According to the pre-survey and statistical analysis, the Cronbach's alpha coefficient of the questionnaire was 0.91.

### For the “Situational Context Design”

The real situational context of REST program is drawn in Table 1.

**Table 1:** The relationship between the situated cognition theory and the REST program.

**Analysis dimension**		**Basic theory**	**Training**	**Training procedure**				**Evaluation of training**	**Training environment**
**Analyze content**		**Situation**	**Cognitive**	**Learning goal**	**Situation**	**Synthesis**	**Collaboration**	**Evaluation**	**Real workload**
Basic	A real s		√	√	√	√	√	√	√
principle	Real activities and responsibilities		√	√	√	√	√	√	√
	Expert performance			√	√		√	√	√
	Multiple roles and diversity		√	√	√	√	√	√	√
	Provide support and guidance			√	√	√	√	√	√
	Collaborative knowledge construction		√	√	√		√	√	√
	Pay attention to reflection		√	√	√	√	√	√	
	Clear expression		√	√	√	√	√		√
	True evaluation		√	√	√	√	√	√	√
Basic features	Context-based action		√	√	√	√	√	√	√
	Legal margin participation			√	√	√	√		
	Community of practice			√	√	√	√		

### For the “Legitimate Peripheral Participation”

Before the outbreak of the epidemic, the public health training received included basic knowledge of infectious diseases (81.58%), putting on and taking off protective clothing and other aseptic operations (84.74%), and hospital infection training (85.26%). Before the outbreak, the public health knowledge and training received came from undergraduate education (83.16%), early resident training (69.47%), online self-study (49.16%), and continuing education (20.53%).

In the REST program, more than 60% indicators of the GP residents' cognitive load on the NASA scale were at a “moderate” level, indicating that the physical and mental workload of the GP residents in the REST program during COVID 19 was at an acceptable level, and the training content, ability improvement, and cognitive load were also fair. In other words, as a GP residents, they were well-prepared for such a pandemic outbreak.

For the questionnaire, The NASA scale showed is as following Table 2. This generally indicated that the intensity of field epidemic prevention and control (training) was tolerable. This also showed that participants were well-trained, prepared, and qualified, with public health knowledge and skills from their former medical schools and through other relevant methods.

**Table 2:** Task cognitive load distribution (from NASA scale) table.

**degree/percentage (%)**	**Mental and**	**Physical burden**	**Time urgency**	**Degree of**	**Task pressure**	**Frustration**
	**cognitive stress**			**effort required**		
Heavy	12.06	20.5	21.9	21.9	11.3	11.3
Middle	62.4	60.9	61	65.2	62.4	62.4
Light	25.54	18.6	17.1	12.9	26.3	26.3

The result of workload is ideal, not too high or too low. And for the data analysis, There was statistical difference (Chi-square estimates, *P* < 0.05) in the cognitive load intensity of participants who participated at different time points after the outbreak of COVID-19 (0~1, 1~3, 3~6 month, later than 6 month), which also shows that the previous and pre-vocational training was relatively sufficient and adapted well to the public health emergency and the epidemic.

### For the “Construction of a Community of Practice”

“Construction of a Community of Practice” call for sociality situatedness. In the REST program, many relevant personnels provided pre-vocational training for prevention and control included undergraduate teachers, hospital-related department teachers, community-based teachers, grassroots government, community staff and hotel staff, senior resident students and other teachers, indicating that a network was initially formed for epidemic prevention and control.

To determine how well-resident doctors would perform if responsible for managing public health disease prevention and control, they were asked, “How well would you perform if you took on the responsibility of prevention and control of an epidemic situation in a community in the future?” The answers were divided into five levels:

- Mid-level: the participants could take on responsibility with guidance and supervision (50%)
- Fair: the participants could accomplish it independently (24.74%)
- Well: the participants could instruct junior or other students (11.58%)
- Very well: the participants would have more constructive suggestions (4.74%)
- Unsure: 8.95%.

Different supervision reflects different relevant learning performance outcomes (26–28).

## Discussion

Whether our education and training for GP resident is suitable or sufficient needs to be verified in practice. The sudden outbreak of the COVID-19 pandemic highlighted the importance of competent community, creating a real worked loaded public health training experience for the GP residents and cognitive load of residents to reveal training implications for how well we do just before the COVID-19 pandemic, during the COVID-19 pandemic and what can we promote for the future (6, 29, 30).

The REST program for GP residents in COVID-19 prevention and control tasks touched workload participation in public health and disease prevention and control and was also a rare, valuable training experience for the residents and research material for medical education.

Based on the Situated learning theory, it concerns the characteristics of learning, such as sociality situatedness, complexity, and competence. Situated learning theory has expanded the nature of learning, which is “from getting to participate.” Legitimate peripheral participation provides an innovative analytical insight into medical education.

From the study, we learned that most GP residents were well-prepared for taking real pandemic prevention and control task during the residents stage, and their public health knowledge and training received came from undergraduate education, early resident training, online self-study and continuing education. As the pandemic prevention and control task need “Construction of a Community of Practice,” many relevant personnel provided pre-vocational training for prevention and control, helping the GP participants to understand how to run the public health control task in the current society. To determine how well-resident doctors would perform if responsible for managing public health disease prevention and control, many shows positive attitude, which reflecting their potential as the “gate keeper.”

The situated learning theory provide the medical education research a framework to design and make assessment for one particular learning content. However, sudden outbreak of the COVID-19 has created a real nature circumstances for public health program design and assessment. Considering the workload result, the REST training mode can be applied in future circumstances. Additionally, the study can be applied to other further study in public health education.

## Advantages of the Study

The sudden outbreak of the COVID-19 pandemic was a rare occurrence in history that allowed for a unique study case. This situated cognition study was naturally designed in a workload training situation in real disease prevention circumstances and included situational context design, legitimate peripheral participation, and construction of a community of practice, which made it unique among other situated cognition studies. The Object Cognitive Load Scale (NASA-TLX scale) (31) were applied to measure the public health training outcome, especially before the COVID-19 pandemic, during the COVID-19 pandemic and in the future.

## Limitations of the Study

Further study need to be followed-up, especially regression discontinuity design (RDD) research method, to reveal the effect of public health prevention training at different stages of the COVID-19 pandemic.

## Data Availability Statement

The original contributions presented in the study are included in the article/Supplementary Material, further inquiries can be directed to the corresponding author/s.

## Ethics Statement

The studies involving human participants were reviewed and approved by West China Hospital Ethics Committee (2020YFQ0011, Sichuan, China). The patients/participants provided their written informed consent to participate in this study.

## Author Contributions

RX designed the research and was major contributor in writing the manuscript. LL and SQ guided the discussion parts. WX discussed the result. All authors participated in the design of the study, contributed to the drafting of the paper, and read and approved the final manuscript.

## Funding

This work was supported by the Funds: Science and Technology Department Project Integrated Health Management and family doctor contract service (2020YFQ0011, Sichuan, China). The study funder aims to improve health conditions in China and make progress in medical education and training.

## Conflict of Interest

The authors declare that the research was conducted in the absence of any commercial or financial relationships that could be construed as a potential conflict of interest.

## Publisher's Note

All claims expressed in this article are solely those of the authors and do not necessarily represent those of their affiliated organizations, or those of the publisher, the editors and the reviewers. Any product that may be evaluated in this article, or claim that may be made by its manufacturer, is not guaranteed or endorsed by the publisher.

## Abbreviations

GP: general practice
REST program: real epidemic situated training.

## References

[1] Roth WM Jornet A. Situated cognition. Wiley Interdiscip Rev Cogn Sci. (2013) 4:463–78. 10.1002/wcs.1242 26304240

[2] Artino AR Jr. It's not all in your head: viewing graduate medical education through the lens of situated cognition. J Grad Med Educ. (2013) 5:177–9. 10.4300/JGME-D-13-00059.1 24404254 PMC3693674

[3] Daniel M Durning SJ Wilson E Abdoler E Torre D. Situated cognition: clinical reasoning and error are context dependent. Diagnosis. (2020) 7:341–42. 10.1515/dx-2020-0011 32562530

[4] Rencic J Schuwirth LWT Gruppen LD Durning SJ. A situated cognition model for clinical reasoning performance assessment: a narrative review. Diagnosis. (2020) 7:227–40. 10.1515/dx-2019-0106 32352400

[5] Smeding A Quinton JC Lauer K Barca L Pezzulo G. Tracking and simulating dynamics of implicit stereotypes: a situated social cognition perspective. J Pers Soc Psychol. (2016) 111:817–34. 10.1037/pspa0000063 27642660

[6] Rencic J Schuwirth LWT Gruppen LD Durning SJ. Clinical reasoning performance assessment: using situated cognition theory as a conceptual framework. Diagnosis. (2020) 7:241–9. 10.1515/dx-2019-0051 32031971

[7] Vogel DHV Jording M Kupke C Vogeley K. The temporality of situated cognition. Front Psychol. (2020) 11:546212. 10.3389/fpsyg.2020.546212 33132954 PMC7550648

[8] Trentini B. Immersion as an embodied cognition shift: aesthetic experience and spatial situated cognition. Cogn Process. (2015) 16(Suppl. 1):413–6. 10.1007/s10339-015-0684-y 26224262

[9] Seamon D. Situated cognition and the phenomenology of place: lifeworld, environmental embodiment, and immersion-in-world. Cogn Process. (2015) 16(Suppl. 1):389–92. 10.1007/s10339-015-0678-9 26224260

[10] Schilhab T Esbensen GL. Socio-cultural influences on situated cognition in nature. Front Psychol. (2019) 10:980. 10.3389/fpsyg.2019.00980 31130897 PMC6509156

[11] Ung COL. Community pharmacist in public health emergencies: quick to action against the coronavirus 2019-nCoV outbreak. Res Social Adm Pharm. (2020) 16:583–6. 10.1016/j.sapharm.2020.02.003 32081569 PMC7129623

[12] Zhang H. Early lessons from the frontline of the 2019-nCoV outbreak. Lancet. (2020) 395:687. 10.1016/S0140-6736(20)30356-1 32059798 PMC7135082

[13] Zhao S Zhuang Z Cao P Ran J Gao D Lou Y. Quantifying the association between domestic travel and the exportation of novel coronavirus (2019-nCoV) cases from Wuhan, China in 2020: a correlational analysis. J Travel Med. (2020) 27:taaa022. 10.1093/jtm/taaa022 32080723 PMC7107546

[14] Artino AR Jr Durning SJ Waechter DM Leary KL Gilliland RW. Broadening our understanding of clinical quality: from attribution error to situated cognition. Clin Pharmacol Ther. (2012) 91:167–9. 10.1038/clpt.2011.229 22261684

[15] Barnett S Jones SC Bennett S Iverson D Robinson L. A virtual community of practice for general practice training: a preimplementation survey. JMIR Med Educ. (2016) 2:e13. 10.2196/mededu.5318 27731864 PMC5041368

[16] Barnett S Jones SC Caton T Iverson D Bennett S Robinson L. Implementing a virtual community of practice for family physician training: a mixed-methods case study. J Med Internet Res. (2014) 16:e83. 10.2196/jmir.3083 24622292 PMC3967123

[17] Barnett S Jones SC Bennett S Iverson D Bonney A. Usefulness of a virtual community of practice and web 2.0 tools for general practice training: experiences and expectations of general practice registrars and supervisors. Aust J Prim Health. (2013) 19:292–6. 10.1071/PY13024 23823006

[18] Catzikiris N Tapley A Morgan S Holliday EG Ball J Henderson K. Maintaining capacity for in-practice teaching and supervision of students and general practice trainees: a cross-sectional study of early career general practices. Aust Health Rev. (2018) 42:643–9. 10.1071/AH16285 28793952

[19] Catzikiris N Tapley A Morgan S Holliday EG Ball J Henderson K. Associations among the advisory working alliance and research self-efficacy within a relational-efficacy framework. J Couns Psychol. (2020) 67:361–70. 10.1037/cou0000389 31580085

[20] Sturman N Parker M Jorm C. Clinical supervision in general practice training: the interweaving of supervisor, trainee and patient entrustment with clinical oversight, patient safety and trainee learning. Adv Health Sci Educ Theory Pract. (2021) 26:297–311. 10.1007/s10459-020-09986-7 32833138

[21] Sturman N Fitzmaurice L Lee C Sheldrake M Ingham G. Good help: a model for providing in-consultation supervision of general practice trainees. Educ Prim Care. (2021) 32:104–8. 10.1080/14739879.2020.1864779 33371787

[22] Ruiz-Rabelo JF Navarro-Rodriguez E Di-Stasi LL Diaz-Jimenez N Cabrera-Bermon J Diaz-Iglesias C. Validation of the NASA-TLX score in ongoing assessment of mental workload during a laparoscopic learning curve in bariatric surgery. Obes Surg. (2015) 25:2451–6. 10.1007/s11695-015-1922-1 26459432

[23] Noyes JM Bruneau DP. A self-analysis of the NASA-TLX workload measure. Ergonomics. (2007) 50:514–9. 10.1080/00140130701235232 17575712

[24] Cao A Chintamani KK Pandya AK Ellis RD. NASA TLX: software for assessing subjective mental workload. Behav Res Methods. (2009) 41:113–7. 10.3758/BRM.41.1.113 19182130

[25] Mansikka H Virtanen K Harris D. Comparison of NASA-TLX scale, modified Cooper-Harper scale and mean inter-beat interval as measures of pilot mental workload during simulated flight tasks. Ergonomics. (2019) 62:246–54. 10.1080/00140139.2018.1471159 29708054

[26] Stensrud TL Mjaaland TA Finset A. Communication and mental health in general practice: physicians' self-perceived learning needs and self-efficacy. Ment Health Fam Med. (2012) 9:201–9. 23997826 PMC3622912

[27] Finomore VS Jr Shaw TH Warm JS Matthews G Boles BD. Viewing the workload of vigilance through the lenses of the NASA-TLX and the MRQ. Hum Factors. (2013) 55:1044–63. 10.1177/0018720813484498 24745198

[28] Morgan S Saltis T Coleman J Tapley A Magin P. Test result audit and feedback (TRAFk) as a supervision method for rational test ordering in general practice training. Aust Fam Physician. (2016) 45:518–22. 27610437

[29] Morrison J Clement T Nestel D Brown J. Perceptions of ad hoc supervision encounters in general practice training: a qualitative interview-based study. Aust Fam Physician. (2015) 44:926–32. 27054214

[30] Rao X Lai J Wu H Li Y Xu X Browning CY. The Development of a Competency Assessment Standard for general practices in China. Front Public Health. (2020) 8:23. 10.3389/fpubh.2020.00023 32154202 PMC7045360

[31] Yurko YY Scerbo MW Prabhu AS Acker CE Stefanidis D. Higher mental workload is associated with poorer laparoscopic performance as measured by the NASA-TLX tool. Simul Healthc. (2010) 5:267–71. 10.1097/SIH.0b013e3181e3f329 21330808

