# Supplementary figures and images for: Real Workload-Situated Training in COVID-19 Prevention of General Practice Residents in China: A Situated Cognition Study
Source: Front Public Health. 2021 Nov 18;9:765402. doi: 10.3389/fpubh.2021.765402 (PMC8637190; doi:10.3389/fpubh.2021.765402)

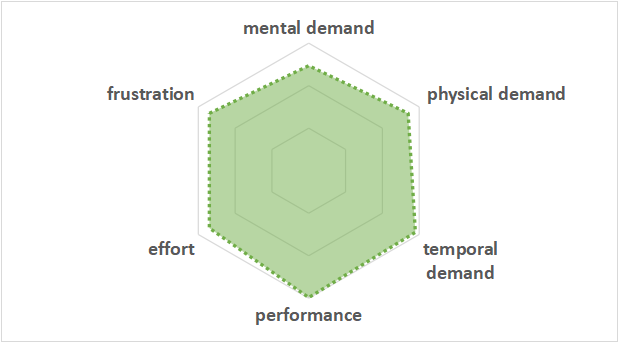

Supplement: Supplementary file 1 [file Image_1.PNG]
